# A Novel Prognostic Signature of Mitophagy-Related E3 Ubiquitin Ligases in Breast Cancer

**DOI:** 10.3390/ijms26041551

**Published:** 2025-02-12

**Authors:** Kangjing Bian, Chihyu Yang, Feng Zhang, Lei Huang

**Affiliations:** Department of Histoembryology, Genetics and Developmental Biology, Key Laboratory of Cell Differentiation and Apoptosis of Chinese Ministry of Education, Shanghai Key Laboratory of Reproductive Medicine, Shanghai Jiao Tong University School of Medicine, Shanghai 200025, China

**Keywords:** breast cancer, prognostic signature, mitophagy, E3 ubiquitin ligase, metabolic, immune

## Abstract

Mitophagy plays a critical role in maintaining mitochondrial quality and cellular homeostasis. But the specific contribution of mitophagy-related E3 ubiquitin ligases to prognoses remains largely unexplored. In this study, we identified a novel mitophagy-related E3 ubiquitin ligase prognostic signature using least absolute shrinkage and selector operator (LASSO) and multivariate Cox regression analyses in breast cancer. Based on median risk scores, patients were divided into high-risk and low-risk groups. Functional enrichment analyses were conducted to explore the biological differences between the two groups. Immune infiltration, drug sensitivity, and mitochondrial-related phenotypes were also analyzed to evaluate the clinical implications of the model. A four-gene signature (ARIH1, SIAH2, UBR5, and WWP2) was identified, and Kaplan–Meier analysis demonstrated that the high-risk group had significantly worse overall survival (OS). The high-risk patients exhibited disrupted mitochondrial metabolism and immune dysregulation with upregulated immune checkpoint molecules. Additionally, the high-risk group exhibited higher sensitivity to several drugs targeting the Akt/PI3K/mTORC1 signaling axis. Accompanying mitochondrial metabolic dysregulation, mtDNA stress was elevated, contributing to activation of the senescence-associated secretory phenotype (SASP) in the high-risk group. In conclusion, the identified signature provides a robust tool for risk stratification and offers insights into the interplay between mitophagy, immune modulation, and therapeutic responses for breast cancer.

## 1. Introduction

Breast cancer is the most common cancer worldwide, with its occurrence being influenced by various risk factors, including genetic and hereditary predisposition [1]. Current therapeutic strategies primarily involve endocrine therapy, anti-HER2 targeting, and chemotherapy. Despite these advancements, breast cancer mortality continues to rise [2,3]. The genomic landscape of diverse types of breast cancer, along with the tumor immune microenvironment (TIME), often leads to different immune infiltration and functionality, which eventually leads to a high recurrence risk and decreased probability of disease-free survival [4]. Overall, better prognostic signatures are needed which can accurately identify risk stratification and deliver personalized treatment.

Mitochondrial autophagy, commonly referred to as mitophagy, is a selective form of autophagy which plays a critical role in maintaining mitochondrial and cellular homeostasis [5]. However, the functions and mechanisms of mitophagy in breast cancer development and progression appear to be complex, as mitophagy acts as both a tumor suppressor and a tumor promoter, depending on the specific context, microenvironment, or cancer stage [6]. Therefore, a deeper understanding of mitophagy-related molecules and their regulatory mechanisms is urgently needed to facilitate the development of more effective therapeutic strategies.

Existing studies have shown that mitophagy can reduce the production of reactive oxygen species (ROS) in tumor cells [7]. In tumor cells with BRCA1 or ULK1 depletion, mitophagy deficiency leads to intracellular ROS accumulation, which triggers NLRP3 inflammasome activation and promotes breast cancer cell growth and metastasis [8]. ROS accumulation also promotes the activation of HIF-1α, which drives the metabolic reprogramming of tumor cells from oxidative phosphorylation to aerobic glycolysis, thereby ensuring energy production during cancer development [9]. Moreover, in the tumor microenvironment (TME), mitophagy plays multifaceted roles, including mediating the metabolic reprogramming of cancer-associated fibroblasts [10], regulating immune cell function [11], and influencing oxidative stress [7].

The mechanism of mitophagy can be broadly categorized into ubiquitin-dependent and ubiquitin-independent pathways [12]. The ubiquitin-dependent pathway is primarily characterized by PINK1/Parkin-mediated mitophagy [13]. In this process, PINK1 accumulates on the outer mitochondrial membrane and phosphorylates ubiquitin, which subsequently recruits Parkin to the mitochondria. This recruitment activates Parkin’s E3 ligase activity, leading to the generation of additional ubiquitin chains. This positive feedback loop facilitates the recruitment of autophagy receptor proteins which recognize ubiquitin chains and interact with LC3 family proteins, ultimately initiating the autophagosome biogenesis machinery [14]. However, the mRNA and protein levels of Parkin are frequently downregulated in breast cancer [15,16], indicating that Parkin is absent when mitophagy is functional. Certain oncoproteins, such as MUC1, trigger cancer development by enhancing mitophagy in a PINK1-dependent manner [17]. This raises the question of whether other E3 ubiquitin ligases are involved downstream of these oncogenic proteins participating in mitophagy, ultimately influencing breast cancer development and metastasis. Furthermore, considering the significance of PINK1/Parkin-mediated mitophagy, it is worthwhile to find out the other E3 ubiquitin ligases which affect survival and investigate the mechanism underlying these potential biomarkers.

**Figure 1:**
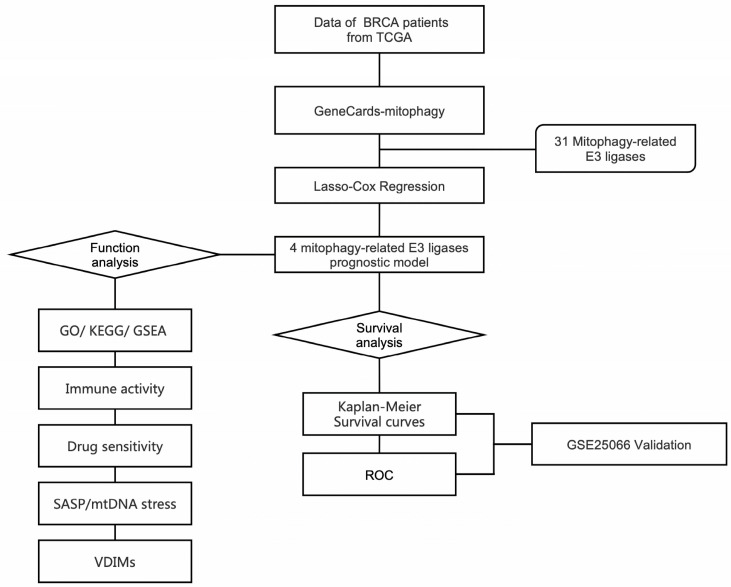
Workflow diagram, showing the flowchart graph of this research.

In this study, RNA sequencing and corresponding clinical data of breast cancer were obtained from The Cancer Genome Atlas (TCGA) and Gene Expression Omnibus (GEO) databases for analysis. A mitophagy-related E3 ligase prognostic model comprising ARIH1, SIAH2, UBR5, and WWP2 was developed to predict patient prognoses. Based on the risk model scores, breast cancer patients were stratified into two groups. Notably, the patients in the high-risk group exhibited poorer overall survival (OS). Our analysis further revealed significant differences in energy metabolism and the immune microenvironment between the two groups. The high-risk group was more sensitive to multiple drugs such as MK-2206, rapamycin, and pictilisib. Data analysis indicates that the process of vesicles derived from the inner mitochondrial membrane (VDIMs), which are responsible for regulating mitochondrial quality, was notably inhibited in the high-risk group. Additionally, SASP driven by mtDNA stress was more pronounced in the high-risk group. In summary, our mitophagy-related E3 ligase risk model shows potential as a prognostic biomarker for guiding personalized treatment strategies in breast cancer patients. The flowchart of this study is shown in Figure 1.

## 2. Results

### 2.1. Construction of Mitophagy-Related E3 Ubiquitin Ligase Prognostic Model

Mitochondrial metabolism plays a crucial role in supporting tumor anabolism [18]. Mitophagy is a critical process for maintaining mitochondrial quality control [19]. Classical mitophagy pathways include PINK1/Parkin-mediated mitophagy and receptor-mediated mitophagy [14]. Receptor-mediated mitophagy has been demonstrated to play a pivotal role in tumorigenesis and contribute to chemotherapy resistance [20,21,22]. Parkin expression is frequently downregulated, mutated, or functionally absent in many tumors, particularly in breast cancer, limiting its role in this context [15,16]. Therefore, elucidating the underlying biological mechanisms of other E3 ubiquitin ligases involved in mitophagy could provide novel perspectives and strategies for breast cancer treatment.

**Figure 2:**
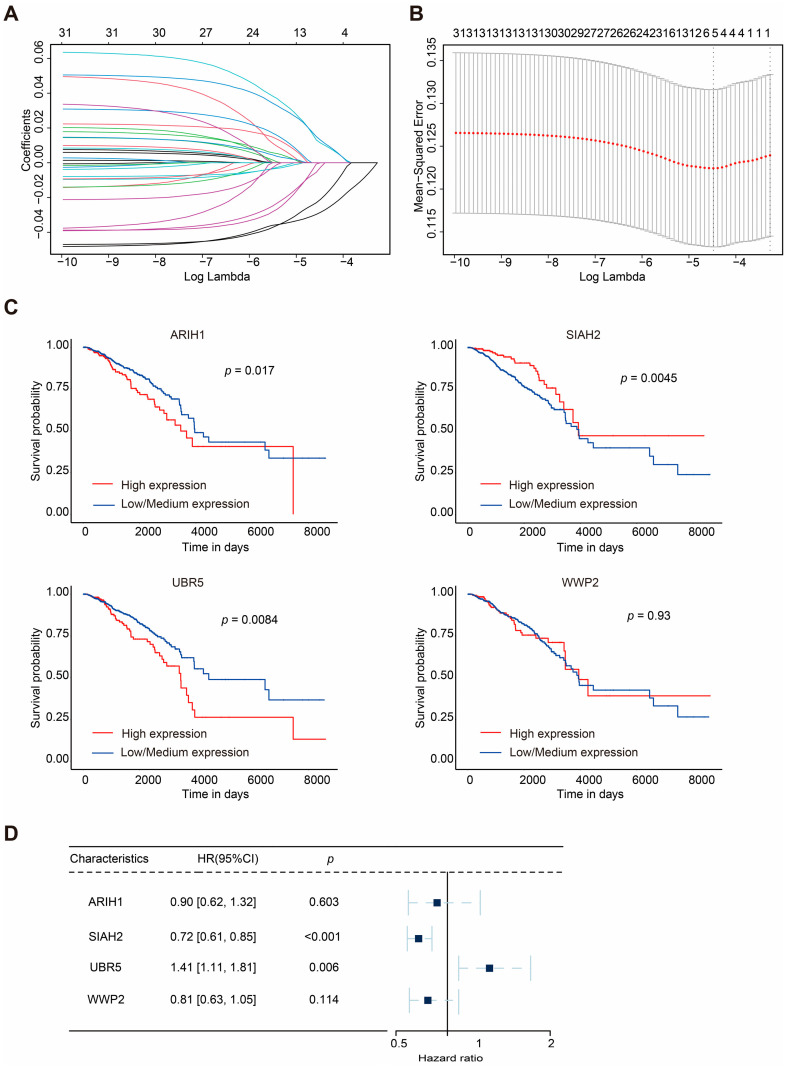
LASSO regression and clinical analyses of prognostic model in breast cancer. (**A**,**B**) Results of LASSO regression analysis. (**C**) Kaplan–Meier survival curves of 4 mitophagy-related E3 ubiquitin ligases (ARIH1, SIAH2, UBR5, and WWP2) in BRCA. (**D**) Multivariate Cox regression analysis of the four selected E3 ubiquitin ligases (ARIH1, SIAH2, UBR5, and WWP2). HRs with 95% CI are displayed. The square represents the HR, and the dashed line indicates 95% CI.

To further investigate mitophagy-related E3 ubiquitin ligases, we retrieved data from GeneCards using the keyword “mitophagy”. Based on the “Description” category, 31 E3 ubiquitin ligases were identified. Combined with transcriptomic data and the corresponding clinical data of BRCA obtained from the TCGA database, statistical analysis of the expression levels of these ligases revealed that most exhibited significant differences (Figure S1A). Subsequently, we utilized the online analysis platform UALCAN (https://ualcan.path.uab.edu) (accessed on 6 December 2024) to evaluate the OS of these mitophagy-related E3 ubiquitin ligases in BRCA patients (Figure S2 and Figure 2C).

The survival analysis indicated that the higher expression of *ARIH1*, *SIAH1*, and *UBR5* was associated with shorter OS in breast cancer patients, whereas the higher expression of *SIAH2* correlated with prolonged survival. Univariate analysis (Figure S1B) revealed that *SIAH2* had a protective effect on prognoses (hazard ratio (HR): 0.76, 95% confidence interval, CI: 0.64–0.89, *p* = 0.00059), whereas *UBR5* was associated with poorer OS in BRCA patients (HR: 1.3, 95% CI: 1.0–1.6, *p* = 0.038).

The gene number was further narrowed down to four according to LASSO-Cox regression analysis (Figure 2A,B). Finally, a prognostic model for BRCA patients, referred to as ASUW, was constructed based on four mitophagy-related E3 ubiquitin ligases: ARIH1, SIAH2, UBR5, and WWP2. Multivariate regression analysis of these four ligases (Figure 2D) revealed that *UBR5* was associated with poor prognoses (HR: 1.41, 95% CI: 1.11–1.81, *p* = 0.006), while *SIAH2* had a favorable effect on prognoses (HR: 0.72, 95% CI: 0.61–0.85, *p* < 0.001). The risk score formula was established as follows: Risk score = ∑ (Coef_i_ × x_i_). Here, x_i_ represents the normalized expression level of the target gene i, and Coef_i_ is the corresponding regression coefficient. These genes and the corresponding coefficients are shown in Table 1.

Assessment of the effectiveness of the risk scores and clinical characteristics (age, stage, T-typing, N-typing, and M-typing) on prognoses through multivariate Cox regression was conducted to determine whether risk scores could independently predict patient survival (Table 2). The results demonstrate that the risk score served as an independent prognostic factor (HR: 2.33, 95% CI: 1.67–3.27, *p* < 0.001).

To compare the expression levels of *ARIH1*, *SIAH2*, *UBR5*, and *WWP2* across different molecular subtypes and stages of BRCA, we analyzed their expression differences using the UALCAN online database (Figure S3, Table S1). Significant differences were identified for *ARIH1*, *SIAH2*, *UBR5*, and *WWP2* in breast cancer stages; *ARIH1* showed significant differences between Normal and Stages 1, 2, 3, and 4 (*p* < 0.05) (Figure S3A), as did *SIAH2* between Normal and Stages 1, 2, 3, and 4 (*p* < 0.05) (Figure S3C), *UBR5* between Normal and Stages 1, 2, and 3 (*p* < 0.05) (Figure S3E), and *WWP2* between Normal and Stages 2 and 3 (*p* < 0.05) (Figure S3G). Similarly, differences in molecular subtypes were observed; *ARIH1* showed significant differences in the Normal versus Luminal and Normal versus TNBC states (*p* < 0.05) (Figure S3B), as did *SIAH2* in the Normal versus Luminal, Normal versus TNBC, Luminal versus HER2-positive, Luminal versus TNBC, and HER2-positive versus TNBC states (*p* < 0.05) (Figure S3D), *UBR5* in the Normal versus Luminal, Normal versus TNBC, and Luminal versus HER2-positive states (*p* < 0.05) (Figure S3F), and *WWP2* in the Normal versus Luminal, Luminal versus TNBC, and HER2-positive versus TNBC states (*p* < 0.05) (Figure S3H).

### 2.2. ASUW Prognostic Model Effectively Predicted Poor Breast Cancer Outcomes

**Figure 3:**
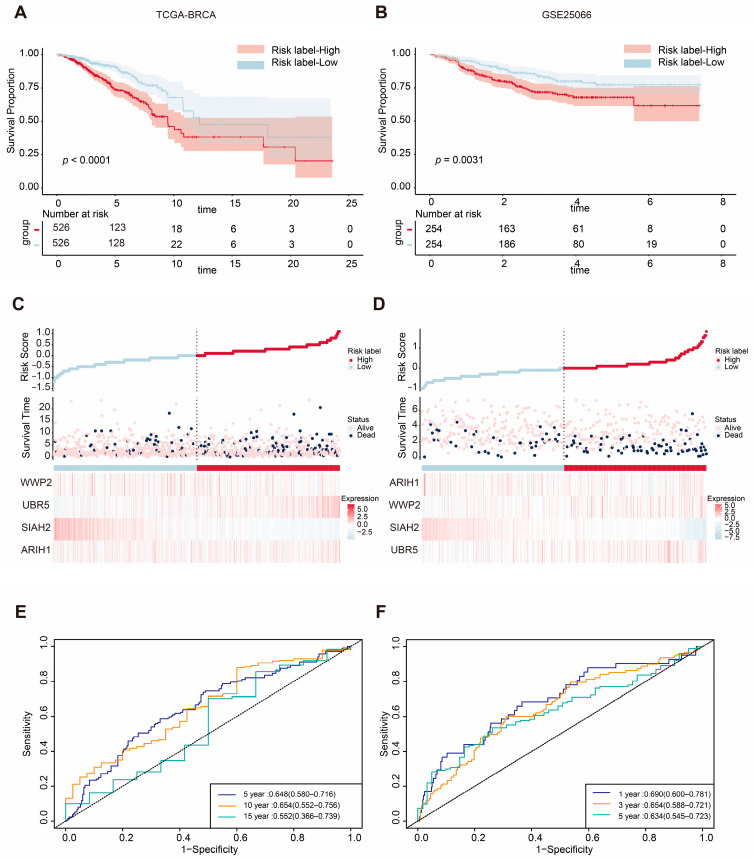
Validation of the ASUW prognostic model in TCGA-BRCA and GSE25066 databases. (**A**,**B**) Kaplan–Meier survival curves for high-risk and low-risk groups in the TCGA-BRCA cohort and GSE25066 cohort, based on the ASUW risk regression model. (**C**,**D**) The distribution of the risk scores, with scatter plots showing whether the samples were alive and heatmaps for the four E3 ubiquitin ligases in the TCGA-BRCA cohort and GSE25066 cohort. Top: Risk scores for each patient, classified as high-risk (red) and low-risk (blue) groups. Middle: Survival time and status (alive or dead). Bottom: Heatmaps of expression levels of the four E3 ubiquitin ligases in two groups. (**E**) ROC curve analysis for 5-, 10-, and 15-year survival predictions in the TCGA-BRCA cohort. (**F**) ROC curve analysis for 1-, 3-, and 5-year survival predictions in the GSE25066 cohort.

To assess the prognostic value of the four mitophagy-related E3 ubiquitin ligase signature, patients in the TCGA-BRCA cohort were classified into high-risk and low-risk groups based on the median risk score. Kaplan–Meier curves revealed that patients in the high-risk group had significantly shorter OS times compared with those in the low-risk group (*p* < 0.0001; Figure 3A). The distribution of risk scores and survival statuses across the two risk subgroups is illustrated in Figure 3C, showing a higher number of deaths in the high-risk group. The heatmap of the genes of the four mitophagy-related E3 ubiquitin ligases is also shown in Figure 3C. To further evaluate the predictive accuracy of the ASUW prognostic model, time-dependent receiver operating characteristic (ROC) curve analysis was performed. The area under the curves (AUCs) for the 5-year, 10-year, and 15-year survival predictions in the TCGA-BRCA cohort were 0.648, 0.654, and 0.552, respectively (Figure 3E), showing the model’s potential for survival prediction.

The efficiency of the prognostic risk model was further validated using the GSE25066 dataset. The same risk formula was applied to the validation cohort from the GEO dataset (GSE25066), and patients were also stratified into high-risk and low-risk groups based on the median risk score. Consistent with the TCGA-BRCA training cohort, Kaplan–Meier survival analysis demonstrated that the patients in the high-risk group had significantly worse overall survival (OS) times compared with those in the low-risk group (*p* = 0.0031; Figure 3B). Additionally, higher risk scores were correlated with poorer survival outcomes (Figure 3D). Notably, the expression patterns of the four mitophagy-related E3 ubiquitin ligases in the GSE25066 validation cohort closely mirrored those observed in the TCGA-BRCA training cohort. In the GSE25066 dataset, the AUCs of the ROC curves for 1-year, 3-year, and 5-year survival predictions were 0.690, 0.665, and 0.634, respectively (Figure 3F). These findings underscore the reliability of the ASUW model as an effective prognostic tool for BRCA patients.

### 2.3. Functional Enrichment Analyses of DEGs Between Two Groups

**Figure 4:**
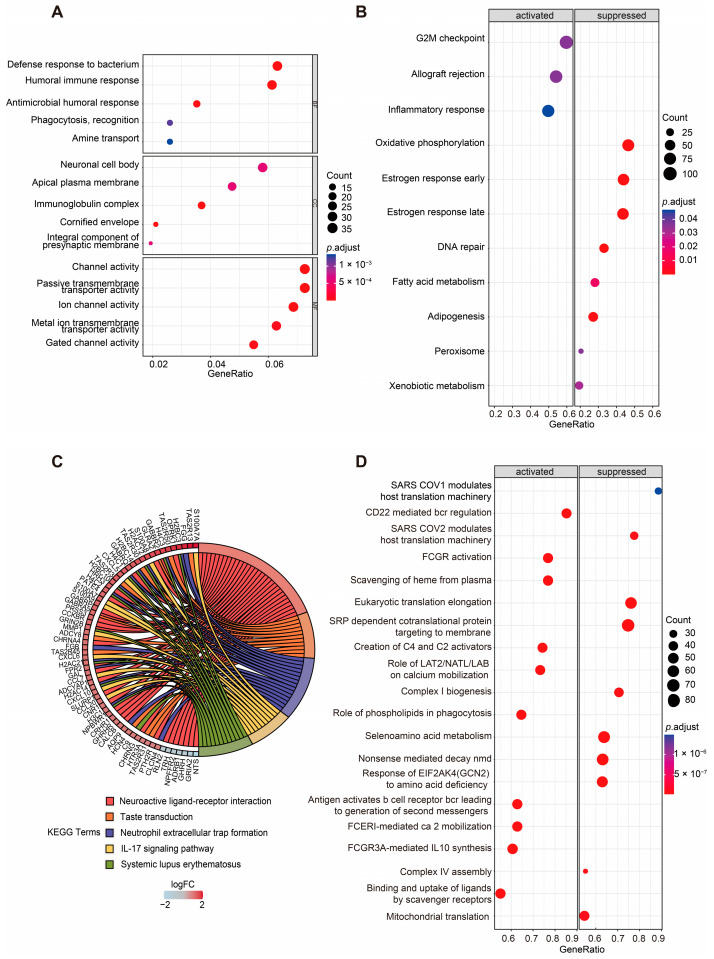
Enrichment analyses of DEGs of two groups. (**A**) GO enrichment analysis of DEGs between the high- and low-risk groups based on the ASUW model. The size of the dots indicates the ratio of genes enriched, while the colors represent the adjusted *p* value. (**B**) GSEA of “Hallmark gene sets” between high-risk and low-risk groups. Significant pathways were categorized as activated (left panel) or suppressed (right panel). The size of the dots represents the ratio of enriched genes, while the color indicates the adjusted *p* value. (**C**) KEGG enrichment analysis of DEGs. A chord diagram demonstrates the associations between the 5 most enriched KEGG pathways and the corresponding genes. The colors of the bands correspond to log (foldchange (FC)) values, showing upregulated (red) and downregulated (blue) genes in the high-risk group. (**D**) GSEA of “Reactome gene sets” between high-risk and low-risk groups. Significant pathways were categorized as activated (left panel) or suppressed (right panel). The size of the dots represents the ratio of enriched genes, while the color indicates the adjusted *p* value.

Given the significantly different OS times observed between the two groups, we sought to further investigate the biological differences between the high-risk and low-risk groups. Differentially expressed genes (DEGs) were identified using the DESeq2 package, and their distribution was visualized through volcano plots to compare the high-risk and low-risk groups (Figure S4A,B, Table S2). Functional enrichment analyses were subsequently performed to explore the biological significance of these DEGs in the two groups. Gene ontology (GO) enrichment analysis revealed that the DEGs annotated to biological processes (BPs) were primarily associated with the defense response to bacterium, humoral immune response, antimicrobial humoral response, phagocytosis, recognition, and amine transport. DEGs annotated to cellular component (CC) categories were predominantly enriched in the neuronal cell body, apical plasma membrane, immunoglobulin complex, cornified envelope, and integral component of the presynaptic membrane. For molecular function (MF), the enriched terms included channel activity, passive transmembrane transporter activity, ion channel activity, metal ion transmembrane transporter activity, and gated channel activity (Figure 4A, Table S3).

Additionally, Kyoto Encyclopedia of Genes and Genomes (KEGG) pathway analysis highlighted the top five enriched pathways as neuroactive ligand–receptor interaction, taste transduction, neutrophil extracellular trap formation, the IL-17 signaling pathway, and systemic lupus erythematosus. The genes enriched in these pathways are visualized in the left half of the circle plot, showing their involvement in these key pathways (Figure 4C, Table S4).

For the Hallmark gene set collection defined by the Molecular Signatures Database (MSigDB), gene set enrichment analysis (GSEA) was conducted to further compare the biological differences between the two groups (Figure 4B, Table S5). The results revealed that the activated pathways in the high-risk group included the G2M checkpoint, allograft rejection, and inflammatory response. In contrast, metabolism-related gene sets such as oxidative phosphorylation, fatty acid metabolism, adipogenesis, peroxisomes, and xenobiotic metabolism were significantly suppressed in the high-risk group. Additionally, DNA repair, early estrogen response, and late estrogen response were also inhibited in the high-risk group.

To gain deeper insights into the metabolic differences between two groups, the Reactome gene set collection from the MSigDB was utilized (Figure 4D, Table S6). The top 10 activated and suppressed pathways, ranked by the normalized enrichment score (NES), were identified. Notably, mitochondria-related pathways, including mitochondrial translation, complex I biogenesis, and complex IV assembly, were significantly suppressed in the high-risk group. What is more, immune-related pathways, such as antigens activating B cell receptors, leading to the generation of second messengers, and FCGR3A-mediated IL-10 synthesis, were activated in the high-risk group.

These findings suggest that the risk score is closely associated with alterations in metabolism and the status of the TME, highlighting the interplay between mitochondrial function, immune responses, and cancer progression.

### 2.4. TIME Comparisons Between Two Groups Under ASUW Prognostic Model

**Figure 5:**
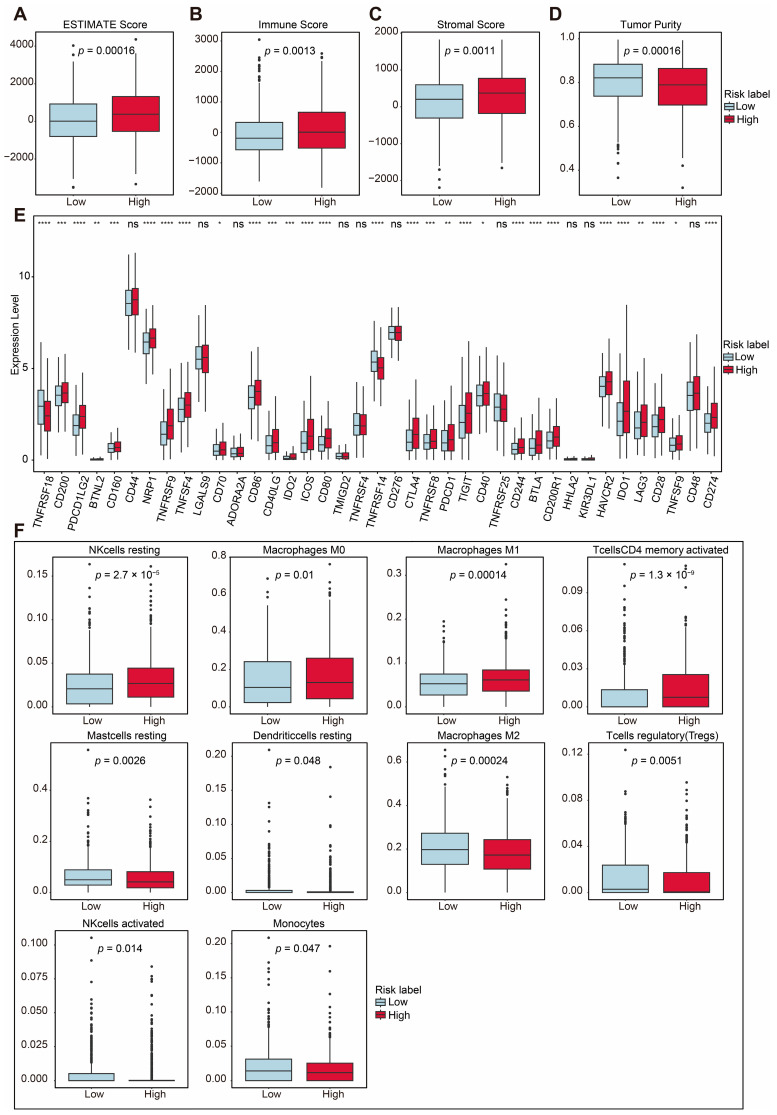
Immune-related analysis between two groups. (**A**) Total ESTIMATE score, indicating tumor purity and immune-stromal component differences (*p* = 0.00016). (**B**) Immune score, indicating immune infiltration differences (*p* = 0.0013). (**C**) Stromal score, indicating stromal content differences (*p* = 0.0011). (**D**) Tumor purity score, indicating tumor purity differences (*p* = 0.00016). (**E**) Comparison of immune checkpoint-related gene expression between the two risk groups. (**F**) Proportions of immune cell types with significant differences between high- and low-risk groups. Statistical significance is indicated as not significant (ns). * *p* < 0.05. ** *p* < 0.01. *** *p* < 0.001. **** *p* < 0.0001.

Mitophagy dysregulation has been shown to play a crucial role in shaping an immunosuppressive tumor microenvironment, thereby facilitating cancer initiation and progression. To further explore the correlation between the risk score and immune microenvironment, we used ESTIMATE to analyze differences in the TIME (Table S7). Patients in the high-risk group exhibited higher ESTIMATE scores (*p* = 0.00016; Figure 5A). Consistently, the high-risk group also showed elevated immune scores (*p* = 0.0013; Figure 5B) and stromal scores (*p* = 0.0011; Figure 5C) compared with the low-risk group. Additionally, tumor purity was significantly lower in the high-risk group (*p* = 0.00016; Figure 5D). These findings suggest that the high-risk group was characterized by a higher level of immune infiltration.

The overexpression of immune checkpoint molecules is frequently linked to immune escape mechanisms [4], and targeting these checkpoints through immunotherapy has been proven to be an effective strategy for BRCA treatment [23]. We further examined the expression of several immune checkpoint-related genes between the high-risk and low-risk groups. Immune checkpoint genes [24,25,26,27,28,29,30,31,32,33], including *PD-L1 (CD274)*, *CTLA-4*, *TIGIT*, *HAVCR2 (TIM3)*, *BTLA*, *CD200*, *CD200R1*, *IDO1*, *IDO2* and *CD160*, were significantly upregulated in the high-risk group (Figure 5E). Among these, PD-L1, CTLA-4, TIM3, LAG3, and TIGIT are key immune checkpoint molecules which play essential roles in regulating immune responses and have been utilized in clinical therapies [34,35,36,37,38].

Using CIBERSORT, we analyzed the proportions of immune cells, as depicted in Figure S5A. The analysis revealed the distribution of 22 immune cell types in breast cancer patients (Table S8). Additionally, there was a certain degree of correlation among these immune cells, as shown in Figure S5B. To further investigate the immune microenvironment, we compared the differences in immune cell proportions between the high-risk and low-risk groups. Out of the 22 immune cell types, 10 exhibited significant changes. The proportions of T cells which were CD4 memory-activated, resting NK cells, M0 macrophages, and M1 macrophages were upregulated in the high-risk group, while the proportions of regulatory T cells (Tregs), activated NK cells, monocytes, M2 macrophages, resting dendritic cells, and resting mast cells were downregulated (Figure 5F). These results highlight distinct immune cell dynamics between the two risk groups, suggesting a potential role of immune cell composition in shaping the tumor microenvironment and influencing prognoses.

### 2.5. Prediction of Sensitive Drugs for BRCA Under ASUW Prognostic Model

**Figure 6:**
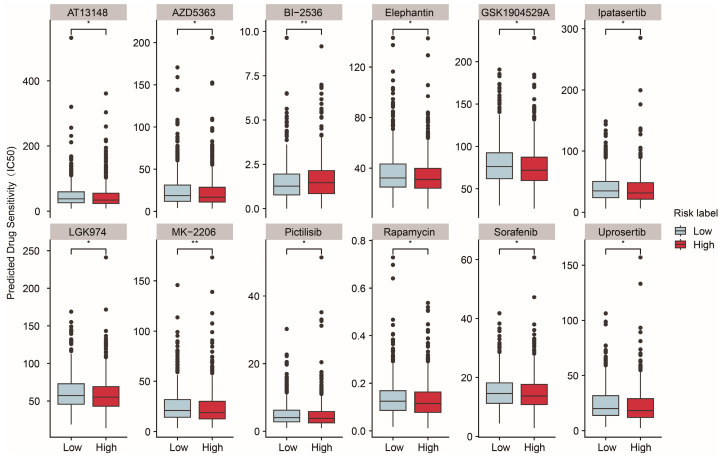
Predicted drug sensitivity differences between two groups. The boxplots depict the predicted drug sensitivity (IC50) of 12 drugs between the high-risk (red) and low-risk (blue) groups as predicted by oncoPredict analysis. Drugs exhibiting statistically significant differences in sensitivity (*p* < 0.05) are shown. The high-risk group displayed higher sensitivity to drugs including MK-2206, pictilisib, rapamycin, sorafenib, GSK1904529A, uprosertib, LGK974, elephantin, AZD5363, ipatasertib, and AT13148. Conversely, the low-risk group demonstrated greater sensitivity to BI-2536. Statistical significance is indicated as * *p* < 0.05. ** *p* < 0.01.

Mitochondria are essential for tumor anabolism, providing critical building blocks, regulating redox and calcium homeostasis, contributing to transcriptional control, and orchestrating cell death [39,40]. Consequently, mitochondrial dysfunction plays a pivotal role in determining the efficacy of innovative anticancer therapies. To further explore the clinical relevance of the ASUW prognostic model for BRCA treatment, the ability of the risk score to predict responses of 198 drugs was analyzed using the “oncoPredict” tool and the GDSC2 database (Table S9). Drug sensitivity, measured by half-maximal inhibitory concentration (IC50) values, was compared between the high- and low-risk groups. The findings revealed that patients in the high-risk group exhibited increased sensitivity to MK-2206, pictilisib, rapamycin, sorafenib, GSK1904529A, uprosertib, LGK974, elephantin, AZD5363, ipatasertib, and AT13148, but decreased sensitivity to BI-2536 (Figure 6). Notably, MK-2206, pictilisib, rapamycin, uprosertib, AZD5363, and ipatasertib are drugs targeting the PI3K/Akt/mTOR signaling pathway. Detailed information for these 12 drugs is provided in Table 3. These results highlight the potential clinical utility of the ASUW prognostic model in guiding personalized therapeutic strategies for BRCA patients.

### 2.6. Analyses of mtDNA Stress, SASP, and VDIM for Two Groups Under ASUW Prognostic Model

**Figure 7:**
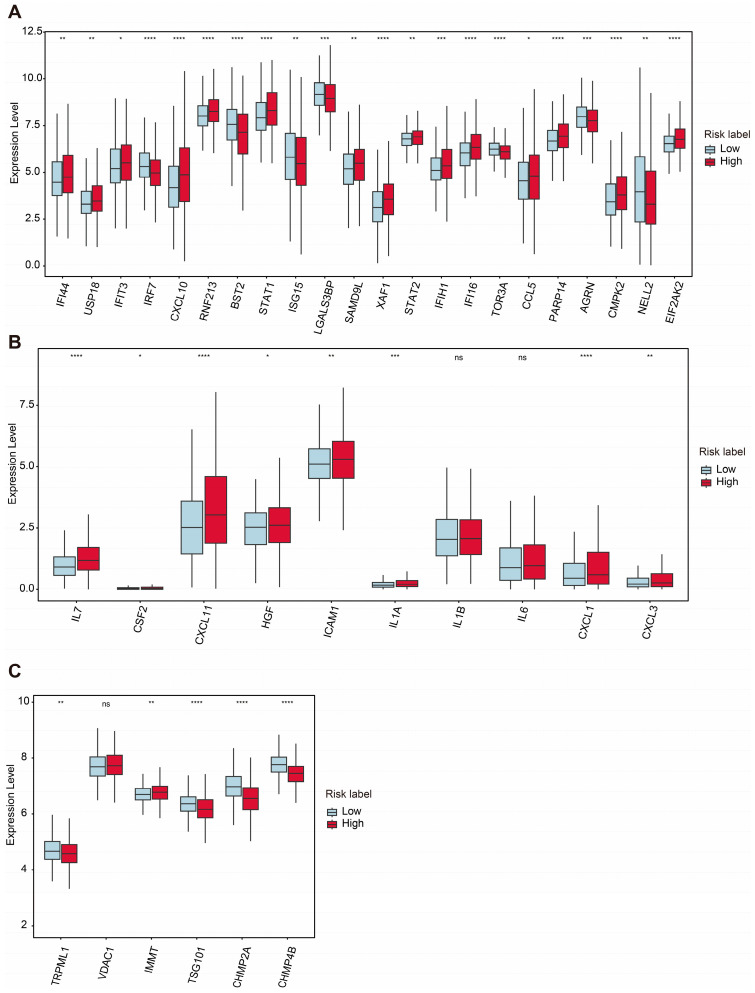
Comparative expression analysis of mtDNA stress and SASP- and VDIM-related genes between two groups. (**A**) Differential expression levels of mtDNA stress-related genes between high- and low-risk groups. (**B**) Differential expression levels of SASP-related genes. (**C**) Differential expression levels of critical proteins involved in the VDIM process. Statistical significance is indicated as not significant (ns). * *p* < 0.05. ** *p* < 0.01. *** *p* < 0.001. **** *p* < 0.0001.

Mitochondrial dysfunction is a hallmark of aging and cellular senescence, significantly influencing the development of the senescence-associated secretory phenotype (SASP), which actively reshapes the tumor microenvironment and facilitates immune evasion by tumors [58,59]. In our analysis, we compared the expression of mtDNA stress signature genes between the high-risk and low-risk groups. Among these genes, *IFI44*, *USP18*, *IFIT3*, *CXCL10*, *RNF213*, *STAT1*, *SAMD9L*, *XAF1*, *STAT2*, *IFIH1*, *IFI16*, *CCL5*, *PARP14*, *CMPK2*, and *EIF2AK2* were significantly upregulated in the high-risk group, while *IRF7*, *BST2*, *STAT1*, *ISG15*, *LGALS3BP*, *TOR3A*, *AGRN*, and *NELL2* were downregulated in the low-risk group (Figure 7A).

Additionally, the expression levels of several SASP-associated genes were examined. Genes such as *IL7*, *CSF2*, *CXCL11*, *HGF*, *ICAM1*, *IL1A*, *CXCL1*, and *CXCL3* were significantly upregulated in the high-risk group, whereas *IL1β* and *IL6* did not show significant differences between the two groups (Figure 7B). These findings highlight the potential role of SASPs in modulating the TME and promoting tumor progression in the high-risk group.

The maintenance of mitochondrial homeostasis relies on the coordinated activity of various quality control mechanisms [60,61]. Among these, mitophagy is a critical process responsible for the removal of damaged mitochondria. Recently, a newly discovered pathway involving vesicles derived from the inner mitochondrial membrane (VDIMs) has been identified specifically as a steady-state mechanism for mitochondrial quality control. The formation of VDIMs is primarily dependent on key molecules, including TRPML1, VDAC1, IMMT, TSG101, CHMP2A, and CHMP4B, which participate in the four main stages of VDIM formation [62].

Our analysis revealed that *TRPML1*, *TSG101*, *CHMP2A*, and *CHMP4B* were significantly downregulated in the high-risk group, while the expression level of *IMMT* was increased in the high-risk group. Interestingly, the expression level of *VDAC1* showed no significant changes between the two groups (Figure 7C). These results suggest a potential impairment in VDIM-related mitochondrial quality control mechanisms in the high-risk group, contributing to mitochondrial dysfunction and its associated pathological effects.

## 3. Discussion

Aberrant regulation of mitophagy has been linked to a variety of diseases, including breast cancer progression. In breast cancer, mitophagy-related signaling molecules demonstrate abnormal expression patterns and play critical roles in biological processes [6]. Mitochondrial function is closely associated with metabolic adaptation, cell proliferation and senescence, and metastasis [21,63,64]. Recent studies have revealed that mitophagy actively reshapes the immune microenvironment, contributing to an immunosuppressive tumor microenvironment which diminishes the efficacy of immunotherapy [65,66,67]. A deeper understanding of the intricate relationship between tumors and mitophagy offers opportunities to propose novel therapeutic strategies. While previous studies have sought to identify mitophagy-related biomarkers at a broad level [68,69], the specific role of mitophagy-related E3 ubiquitin ligases in predicting breast cancer prognoses remains largely unexplored.

This study identified a novel mitophagy-related E3 ubiquitin ligase signature which can effectively predict prognosis in BRCA patients. The risk score derived from this model was significantly associated with metabolism dysfunction, the immune microenvironment, and drug sensitivity, highlighting its potential as a prognostic biomarker and therapeutic target.

After constructing the prognostic model consisting of ARIH1, SIAH2, UBR5, and WWP2, TCGA-BRCA patients were stratified into two groups based on the median risk score. The expression levels of four E3 ligases were all significantly changed compared with normal tissue, especially in terms of the luminal type of BRCA. Notably, ARIH1 is a key E3 ubiquitin ligase involved in mitophagy via a Parkin-independent pathway, particularly in response to mitochondrial damage, ultimately enhancing chemotherapy sensitivity [70]. ARIH1 promotes anti-tumor immunity by targeting PD-L1 for proteasomal degradation in breast cancer. Mechanistic findings suggest that inhibition of EGFR activates GSK3α by suppressing AKT activity, which subsequently promotes the phosphorylation of PD-L1, leading to ARIH1-mediated ubiquitination and proteasome-mediated degradation [71]. Our findings indicate that a low level of *ARIH1* expression is associated with higher PD-L1 (CD274) expression and thus a high-risk of a poor prognosis. The exact mechanism linking ARIH1 and mitophagy, which could influence the effectiveness of immunotherapy and chemotherapy, remains unclear.

Currently, no direct studies have confirmed whether SIAH2, UBR5, or WWP2 promote or inhibit mitophagy. However, knocking out SIAH2 has been shown to prevent the degradation of mitochondrial NCX3, regulate mitochondrial fission and fusion dynamics, and restore mitochondrial function in hypoxic neurons [72]. SIAH1, a member of the same family as SIAH2, has been reported to promote mitophagy through the PINK1-synphilin-1-SIAH1 signaling axis, contributing to the progression of Parkinson’s disease [73]. Conversely, SIAH3, which lacks catalytic activity, has been shown to inactivate PINK1 and accumulate on damaged mitochondria, thereby inhibiting mitophagy [74]. In vitro experiments showed that SIAH2 silencing in MCF7 cells led to resistance to anti-estrogen ICI164.384 treatment compared with mock-silenced cells. In primary breast cancer patients treated with adjuvant tamoxifen, SIAH2 was associated with improved metastasis-free survival (HR = 0.73; *p* = 0.005) [75]. However, there is no evidence indicating that SIAH2 contributes to chemoresistance through mitophagy or mitochondrial quality control. Our results show that low levels of *SIAH2* expression are related to disordered mitochondrial metabolism and high risk for a poor prognosis.

UBR5 is frequently amplified and overexpressed in many cancer types, especially in human breast cancer [76]. In ER+ breast cancer, UBR5 overexpression induces tamoxifen resistance in vitro, whereas UBR5 knockdown enhances tamoxifen sensitivity. Mechanistic investigations revealed that UBR5 overexpression led to the upregulation of β-catenin expression and activity [77]. UBR5 also plays a key role in IFN-γ-induced PDL1 transcription in TNBC and finally promotes tumor immune evasion [78]. Apart from this, overexpression of UBR5 promotes tumor growth in gallbladder cancer via the PTEN/PI3K/Akt signal pathway [79]. In colorectal carcinoma, UBR5 knockdown inhibits cancer cell proliferation by reducing the expression of PYK2, which subsequently downregulates oxidative phosphorylation and suppresses metabolic reprogramming [80]. We found that in the high-risk group, which was sensitive to PI3K/Akt/mTOR-targeted drugs, the expression levels of both *UBR5* and *PD-L1* were high. Further studies on the mechanisms involving mitophagy, PI3K/Akt, and UBR5 are needed for the therapeutic development of breast cancer.

Meanwhile, studies have suggested that WWP2 may play a role in the degradation of mitophagy receptors, such as NDP52, OPTN, and SQSTM [81]. However, direct evidence linking WWP2 to the regulation of mitophagy through this pathway is currently lacking. A recent study showed that WWP2 functions as an E3 ligase for the Notch1 intracellular domain (NICD1) at K1821 and acts as a suppressor of breast cancer metastasis. WWP2 expression is downregulated in advanced breast cancer tissues. Moreover, breast cancer tissues with higher WWP2 expression exhibit decreased Ki67 and NICD1 signals. Low WWP2 expression is associated with unfavorable overall survival in breast cancer patients [82]. Our results also showed a negative correlation between low *WWP2* levels and the high-risk group, which exhibited activation of the G2/M checkpoint.

Considering these findings, further research is urgently needed to elucidate the roles of ARIH1, SIAH2, UBR5, and WWP2 in modulating mitophagy, mitochondrial homeostasis, and drug resistance in cancer.

As the Kaplan–Meier survival analysis demonstrated a significant association between a high-risk group and poor prognoses, we further examined the biological differences between the high- and low-risk groups. The GSEA results revealed that pathways such as the G2M checkpoint and inflammatory response were upregulated in the high-risk group. These pathways are well-known contributors to poor survival rates, reduced drug responsiveness, and a more immunosuppressive tumor microenvironment [83,84]. Additionally, estrogen response (early and late) pathways were downregulated in the high-risk group. Given the established association between estrogen receptor positivity and lower recurrence rates [23], the downregulated estrogen response further explains the poor prognoses observed in the high-risk group.

GO enrichment analysis highlighted both immune dysregulation and apparent ion channel activity dysregulation in the high-risk group. It is worth noting that ion channel activity is critical for maintaining lysosomal and mitochondrial homeostasis [85,86]. The proper functioning of mitochondria largely relies on the electrochemical proton gradient. A key component of this gradient, the inner mitochondrial membrane potential, is tightly regulated by ion transport across mitochondrial membranes [87]. These results indicate that mitophagy has a connection with ion channel activity.

As we observed that pathways related to the defense response to bacterium, antimicrobial humoral response, and phagocytosis recognition were all upregulated in the high-risk group according to the GO enrichment results, we hypothesized that microbes hijacking tumor cells may act as drivers of tumor cell seeding. Supporting this notion, the upregulation of neutrophil extracellular trap formation and the IL-17 signaling pathways, as revealed by KEGG enrichment analysis, aligns with findings from previous studies [88].

Recently, accumulating evidence has shown that human tumors harbor substantial amounts of viable commensal microbiota [89,90,91]. For instance, a recent study demonstrated that a gut microbial metabolite activates mitophagy to regulate microglia-mediated neuroinflammation and mitigate the progression of herpes simplex encephalitis [92]. Additionally, *Escherichia coli* Nissle 1917 was found to inhibit mitophagy and ameliorate mitochondrial damage by promoting IL-22 expression in polycystic ovary syndrome mice [93]. These findings suggest that microbiota can influence the immune environment through mitophagy, raising the intriguing possibility of a connection between tumor-resident intracellular microbiota and mitophagy. Further investigation into this relationship could provide valuable insights, potentially uncovering drug targets such as E3 ubiquitin ligases which regulate mitophagy, thereby offering novel therapeutic strategies for cancer treatment.

The enrichment analysis indicated immune dysregulation in the high-risk group, which was further supported by the ESTIMATE analysis showing higher immune infiltration accompanied by an immunosuppressive status in the high-risk group. Dysregulation of mitophagy has been demonstrated to play a significant role in establishing an immunosuppressive microenvironment, thereby fostering cancer initiation and progression [94]. Studies have revealed that mitophagy shapes the tumor microenvironment by regulating the PD-1/PD-L1 signaling pathway. Impaired mitophagy weakens antitumor immune responses and facilitates immune evasion, as observed in hepatocellular carcinoma. Parkin, an E3 ubiquitin ligase, has been shown to induce the ubiquitination and degradation of PD-1, ultimately modulating antitumor immunity through the PD-1/PD-L1 pathway [95].

Moreover, defective mitophagy results in the accumulation of damaged mitochondria, leading to increased reactive oxygen species production and the release of mitochondrial DNA. Mitochondrial homeostasis is maintained by the removal of damaged cristae via vesicles derived from the inner mitochondrial membrane (VDIMs) at a steady status. The significant downregulation of *TRPML1*, *TSG101*, *CHMP2A*, and *CHMP4B* in the high-risk group suggests impaired VDIM-mediated mitochondrial quality control, potentially contributing to mtDNA stress and the activation of senescence-associated secretory phenotypes. These changes promote immunosuppressive signaling and recruit tumor-promoting immune cells, such as myeloid-derived suppressor cells and regulatory T cells (Tregs) [94]. Intriguingly, a recent study developed a mitochondrial localized in situ self-assembly system which shifts mitophagy from a pro-survival mechanism to a pro-death mechanism, transforming the immunosuppressive tumor microenvironment into an immunostimulatory one [96]. Overall, dysregulated mitophagy disrupts the balance of the immune and metabolic microenvironments, thereby creating conditions which support cancer development and progression.

Notably, the enhanced drug sensitivity to many PI3K/Akt/mTOR inhibitors in the high-risk group suggests that these patients may benefit from targeted therapies, offering a potential strategy for improving treatment outcomes [97]. Recent studies have highlighted the interplay between the PI3K/Akt/mTOR signaling pathway and mitophagy in maintaining mitochondrial homeostasis. For example, inhibition of SGLT1 was found to increase mitophagy and restore mitochondrial function by suppressing the PI3K/Akt pathway in radiation-induced intestinal injury, underscoring the critical role of this pathway in modulating mitophagy [98]. Similarly, metformin treatment, while alleviating ultraviolet radiation-induced cellular damage, regulated the PI3K/Akt/mTOR pathway, along with reduced mitochondrial oxidative stress and decreased mitophagy [99]. These findings suggest that dysregulation of the PI3K/Akt/mTOR signaling pathway not only affects mitochondrial quality control via mitophagy but also exacerbates oxidative stress, thereby establishing a connection to finding therapeutic strategies for conditions involving mitochondrial dysfunction. However, the specific role of mitophagy-related E3 ubiquitin ligases in regulating the crosstalk between PI3K/Akt/mTOR signaling and mitophagy remains an area which needs further investigation.

Systemic therapy for breast cancer is commonly guided by classical immunohistochemistry markers such as the estrogen receptor, progesterone receptor, and HER2, which are conventionally used for breast cancer prognosis and classification [3]. The prognostic signature we identified is associated with mitophagy. The four genes identified in this study may inspire new directions for understanding the function of mitophagy in drug resistance and immune dysregulation in breast cancer. Given that many drugs targeting mitophagy have been applied for clinical purposes [14], stratification based on this signature could complement existing major classifications and facilitate the use of mitophagy-related targeted therapies. Furthermore, the mitophagy-related E3 ligase signature does not conflict with the current major classifications of breast cancer. Therefore, this finding provides a new perspective on breast cancer, from prognosis to personalized therapeutic strategies.

While this study leveraged comprehensive transcriptomic datasets to construct and validate the prognostic model, further experimental validation is necessary to confirm the functional roles of the identified E3 ubiquitin ligases. Additionally, incorporating multi-omics data, such as proteomics and metabolomics, could provide deeper insights into the underlying mechanisms.

In conclusion, this study sheds light on the intricate relationship between mitophagy, the tumor microenvironment, and breast cancer prognoses. The identified mitophagy-related E3 ligase signature holds promise as a prognostic biomarker and therapeutic target, paving the way for personalized treatment strategies for breast cancer.

## 4. Materials and Methods

### 4.1. Data Collection and Preparation of BRCA

The gene expression profiles and clinical data for breast cancer were obtained from the TCGA database via UCSC Xena. After excluding samples lacking corresponding clinical information, 111 normal samples and 1052 tumor samples with both clinical and expression data were included for further analysis.

For external validation, the dataset GSE25066 was downloaded from the Gene Expression Omnibus (GEO). GSE25066 was generated using the GPL96 platform (https://www.ncbi.nlm.nih.gov/geo/query/acc.cgi?acc=GPL96, accessed on 9 February 2025), comprising 508 tumor samples of breast cancer patients. Probe identifiers were mapped to gene symbols according to the platform annotation for the GSE25066 dataset. Patients from the TCGA dataset were used for the training cohort, while the GSE25066 dataset was used for external validation.

### 4.2. Mitophagy-Related Gene Collection

A total of 5177 mitophagy-related genes were identified from GeneCards. Based on their descriptions, 31 mitophagy-related E3 ubiquitin ligases were selected for further analysis.

### 4.3. Univariate Cox Regression Analysis

Univariate Cox regression analysis was performed to evaluate the prognostic significance of individual mitophagy-related E3 ubiquitin ligases. The hazard ratio and corresponding 95% confidence interval were calculated for each gene to evaluate its association with overall survival in BRCA.

### 4.4. Construction of a Prognostic Model

The 31 mitophagy-related E3 ubiquitin ligases were further refined using least absolute shrinkage and selection operator regression analysis, conducted with the R package “glmnet” (4.1.8), to identify key genes for constructing a multivariate Cox regression model. The regression coefficients (Coef_i_) for each selected gene included in the prognostic model were determined through multivariate Cox regression analysis. Subsequently, the risk score for each patient in the TCGA dataset was calculated based on the formula derived from the multivariate Cox model. Patients were stratified into low-risk and high-risk groups, using the median risk score as the cut-off value. The same regression coefficient was used to calculate the risk scores of patients in the GSE25066 dataset. Patients were divided into low-risk and high-risk groups according to the median risk score.

### 4.5. Overall Survival Analysis

Survival curves were generated using the Kaplan–Meier method, and the log-rank test was applied to assess survival differences between the two groups. Statistical significance was defined as a *p* value <0.05. Additionally, the R package “timeROC ” (0.4) was utilized to generate time-dependent receiver operating characteristic curves, allowing for evaluation of the prognostic model’s predictive performance. The area under the curve values were calculated to quantify the accuracy of the model in predicting survival probabilities over specific time intervals. The R package “ggrisk” (1.3) was used to generate the risk score distribution, survival status, and heatmap. This risk factor association plot provided an integrated view of the relationship between risk scores, survival status, and gene expression, enabling a comprehensive assessment of the prognostic model.

### 4.6. DEG Identification

Differentially expressed genes (DEGs) between the two groups were identified through the R package “Deseq2” (1.38.3). |Log (2) fold change| > 1 and an adjusted *p* < 0.05 were the criteria for defining DEGs. The R packages “pheatmap” (1.0.12) and “ggplot2” (3.4.4) were employed to generate volcano maps and heatmaps to visualize the DEGs.

### 4.7. Gene Ontology (GO) and Kyoto Encyclopedia of Genes and Genomes (KEGG) Analyses

To better understand the underlying differences between two groups, enrichment analyses, including gene ontology (GO) and the Kyoto Encyclopedia of Genes and Genomes (KEGG), were conducted using the R packages “clusterProfiler” (4.6.2), “org.Hs.eg.db” (3.16.0), “enrichplot” (1.18.4), and “ggplot2” (3.4.4). A significance threshold of *p* < 0.05 was applied. The GO analysis categorized the enriched terms into three domains—biological process (BP), cellular component (CC), and molecular function (MF)—providing insights into the biological roles and mechanisms associated with the DEGs. These analyses were instrumental in uncovering the functional annotations and pathway associations of the identified DEGs.

### 4.8. Gene Set Enrichment Analysis (GSEA)

The R package “clusterProfiler” (4.6.2) was used to perform GSEA to evaluate the functional pathway differences between the two groups. Enrichment analysis was conducted using the Hallmark and Reactome gene sets from the Molecular Signatures Database. Pathways with Benjamini–Hochberg (BH) adjusted *p* values <0.05 were considered significantly enriched. The results were visualized using the R package “enrichplot”, providing a presentation for the significantly enriched pathway.

### 4.9. ESTIMATE Analysis

The ESTIMATE algorithm was employed to evaluate the tumor microenvironment’s stromal and immune components through the R package “estimate” (1.0.13). This method quantifies stromal and immune cell infiltration within tumor samples based on gene expression profiles, providing four key scores—the stromal score, immune score, tumor purity, and ESTIMATE score—which collectively reflect tumor purity. Gene expression profiles were input into the ESTIMATE algorithm to calculate the stromal and immune scores for each sample. These scores were used to compare the tumor microenvironment characteristics between the high-risk and low-risk patient groups. The results were visualized using the R package “ggplot2” to highlight differences in the tumor microenvironment features between the groups.

### 4.10. Evaluation of Drug Sensitivity

The R package “oncoPredict” (1.2) is a powerful tool for predicting clinical drug response in patients using gene expression profiles. It constructs statistical models based on gene expression profiles and drug sensitivity data from cell lines in Sanger’s Genomics of Drug Sensitivity in Cancer (GDSC) database [100]. Using the “oncoPredict” package, we estimated the half-maximal inhibitory concentration (IC50) values for 198 drugs in both the high-risk and low-risk patient groups, enabling a comprehensive comparison of the drug sensitivity between the groups.

### 4.11. Statistical Analysis

Statistical analyses were conducted using R Studio (version 4.2.3). For comparisons between two groups with continuous variables, an independent *t*-test was employed to assess the statistical significance of variables with a normal distribution. For non-normally distributed variables, the Wilcoxon rank-sum test was used. A *p* value < 0.05 was considered statistically significant.

## Figures and Tables

**Table 1:** The information for 4 mitophagy-related E3 ubiquitin ligases.

Gene Symbol	Gene ID	Full Name	Classes	Risk Coefficient
*ARIH1*	25820	Ariadne RBR E3 Ubiquitin Protein Ligase 1	RING-IBR-RING (RBR)	−0.100073572
*SIAH2*	6478	Siah E3 Ubiquitin Protein Ligase 2	Really interesting new gene (RING)	−0.333969422
*UBR5*	51366	Ubiquitin Protein Ligase E3 Component N-Recognin 5	Homologous to the E6AP carboxyl terminus (HECT)	0.34620336
*WWP2*	11060	WW Domain Containing E3 Ubiquitin Protein Ligase 2	Homologous to the E6AP carboxyl terminus (HECT)	−0.204703988

**Table 2:** Multivariate Cox regression analysis integrating risk scores with clinical characteristics.

Characteristics	HR (95% CI)	*p* Value
Stage (vs. Stage I)		
Stage II	1.21 [0.51, 2.83]	0.666
Stage III	1.81 [0.58, 5.66]	0.304
Stage IV	9.43 [0.76, 117.34]	0.081
Tumor (vs. T1)		
T2	1.22 [0.65, 2.27]	0.538
T3	1.18 [0.53, 2.62]	0.678
T4	1.61 [0.65, 4.00]	0.306
Lymph Node (vs. N0)		
N1	1.53 [0.94, 2.50]	0.090
N2	1.71 [0.75, 3.90]	0.206
N3	1.67 [0.74, 3.76]	0.212
Metastasis (vs. M0)		
M1	0.71 [0.09, 5.95]	0.756
Age	1.03 [1.02, 1.05]	<0.001
Risk Score	2.33 [1.67, 3.27]	<0.001

**Table 3:** Detailed information for 12 sensitivity drugs.

Drug Name	Target	Application	Reference	IC50 (High vs. Low)
MK-2206	Akt	Lung cancer, breast cancer, Colorectal cancer	[41,42]	down
Pictilisib	PI3K	Breast cancer, lung cancer	[43,44]	down
Rapamycin	mTORC1	Breast cancer, kidney cancer	[45,46]	down
Sorafenib	VEGFR, PDGFR, RAF kinases	Hepatocellular carcinoma, gastric cancer	[47,48]	down
BI-2536	PLK1	Breast cancer, lung cancer	[49,50]	up
GSK1904529A	IGF-1R	Breast cancer	[51]	down
Uprosertib	Akt	Colorectal cancer	[52]	down
LGK974	Porcupine	Colorectal cancer	[53]	down
Elephantin	Unclear	Further investigation is required	Unclear	down
AZD5363	Akt	Breast cancer	[54]	down
Ipatasertib	Akt	Breast cancer, prostate cancer	[55,56]	down
AT13148	AGC family kinases	Pancreatic ductal adenocarcinoma	[57]	down

## Data Availability

The datasets of this article were generated from the TCGA database and GEO database.

## Supplementary Materials

The following supporting information can be downloaded at: https://www.mdpi.com/article/10.3390/ijms26041551/s1.

## Abbreviations

The following abbreviations are used in this manuscript:

SASP	Senescence-associated secretory phenotype
TME	Tumor microenvironment
BRCA	Breast cancer
TIME	Tumor immune microenvironment
TCGA	The Cancer Genome Atlas
GEO	Gene Expression Omnibus
OS	Overall survival
VDIMs	Vesicles derived from the inner mitochondrial membrane
GSEA	Gene set enrichment analysis
LASSO	Least absolute shrinkage and selector operator
ROC	Receiver operating characteristic
AUC	Area under the curve
DEG	Differentially expressed gene
GO	Gene ontology
BP	Biological processes
CC	Cellular component
MF	Molecular function
KEGG	Kyoto Encyclopedia of Genes and Genomes
GSEA	Gene set enrichment analysis
MSigDB	Molecular Signatures Database
HR	Hazard ratio
CI	Confidence interval
KM	Kaplan–Meier
