# Supplementary material for: A Novel Prognostic Signature of Mitophagy-Related E3 Ubiquitin Ligases in Breast Cancer
Source: Int J Mol Sci. 2025 Feb 12;26(4):1551. doi: 10.3390/ijms26041551 (PMC11855622; doi:10.3390/ijms26041551)
Supplement: Supplementary file 1 [file ijms-26-01551-s001.zip › supplementary.pdf]

# A Novel Prognostic Signature of Mitophagy-Related E3 Ubiquitin Ligases in Breast Cancer

**Kangjing Bian, Chihyu Yang, Feng Zhang * and Lei Huang ***

Department of Histoembryology, Genetics and Developmental Biology, Key Laboratory of Cell Differentia-tion and Apoptosis of Chinese Ministry of Education, Shanghai Key Laboratory of Reproductive Medicine, Shanghai Jiao Tong University School of Medicine, Shanghai, China;
kangjing@sjtu.edu.cn;
nevaehbsb@sjtu.edu.cn ;
leihuang@shsmu.edu.cn
*Correspondence: fzhang20@sjtu.edu.cn; leihuang@shsmu.edu.cn

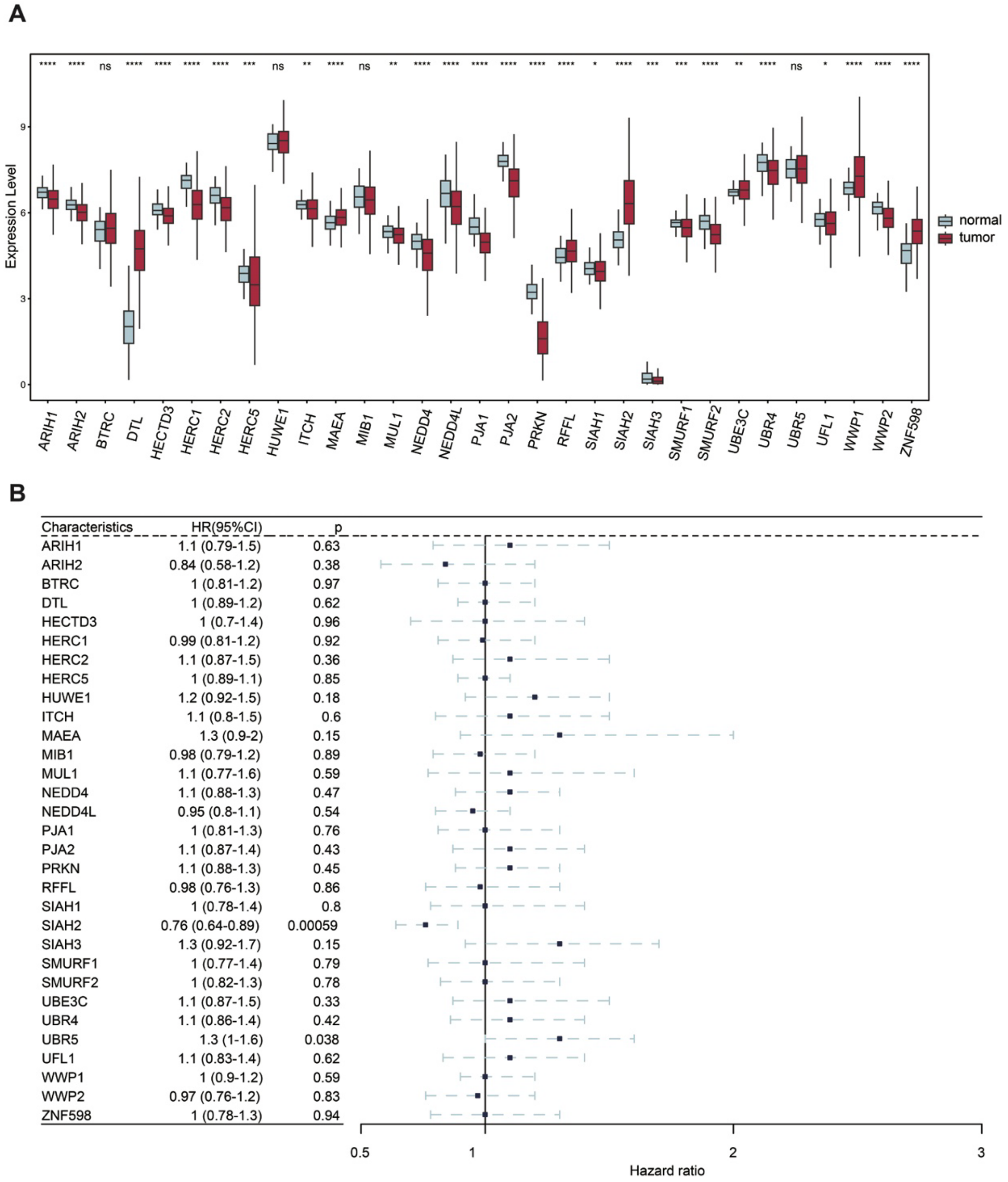


**Figure S1.** Comparative expression and univariate Cox regression analyses of mitophagy-related E3 ubiquitin ligases in Breast Cancer. (A) Comparative expression analysis of 31 mitophagy-related E3 ubiquitin ligases between breast cancer and normal samples. The red boxplots represent tumor samples, while the blue boxplots represent normal samples. Statistical significance is indicated as: ns (not significant), *$p < 0.05$, **$p < 0.01$, ***$p < 0.001$, ****$p < 0.0001$. (B) Univariate Cox regression analysis of 31 mitophagy-related E3 ubiquitin ligases. HRs and 95% CI are shown, with statistical significance denoted by p-values.

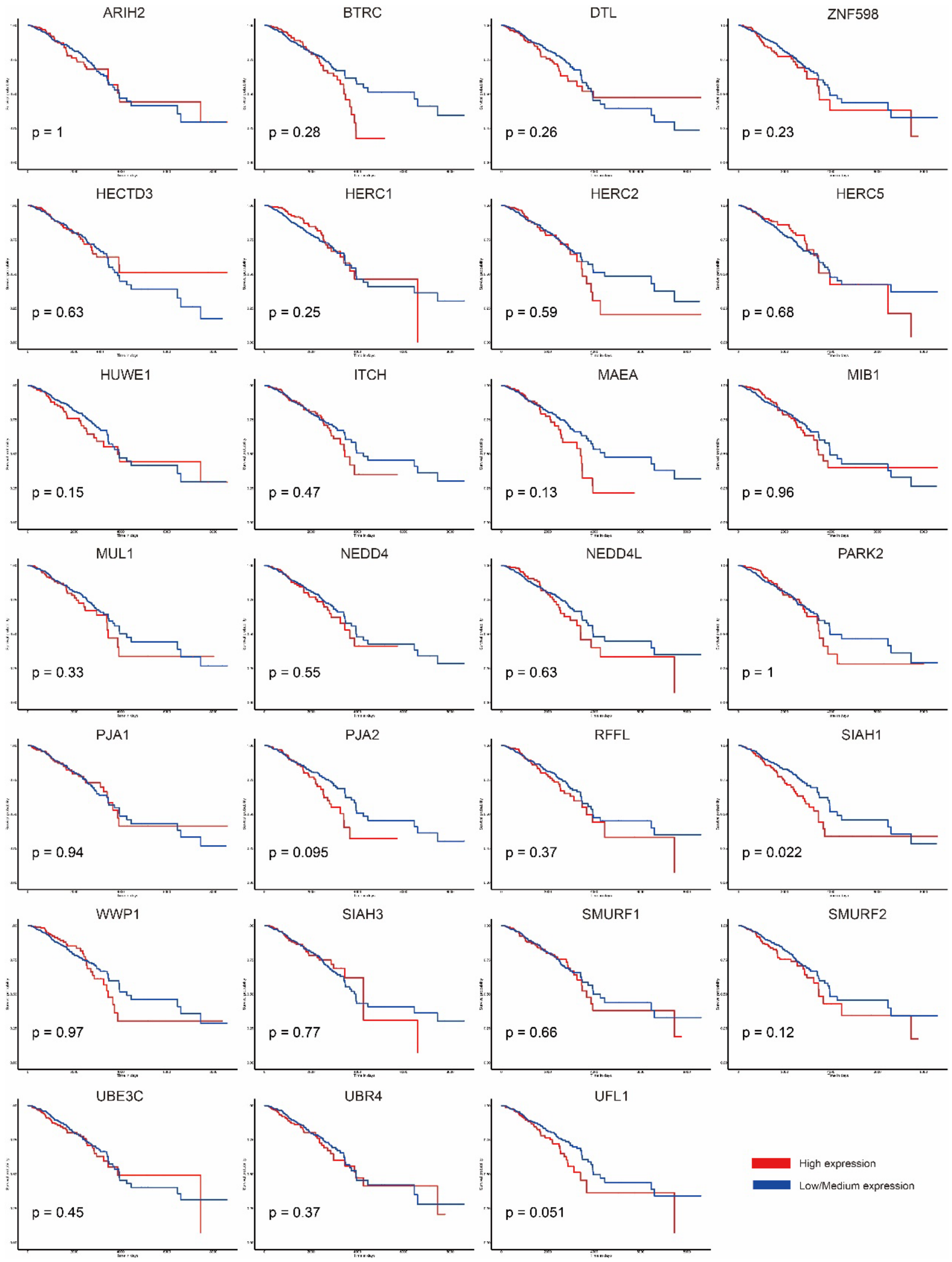


**Fig S2.** Kaplan-Meier survival curves of 27 mitophagy-related E3 ubiquitin ligases in BRCA. High expression groups are shown in red, and low/medium expression groups are shown in blue. Statistical differences in survival between the groups were evaluated.

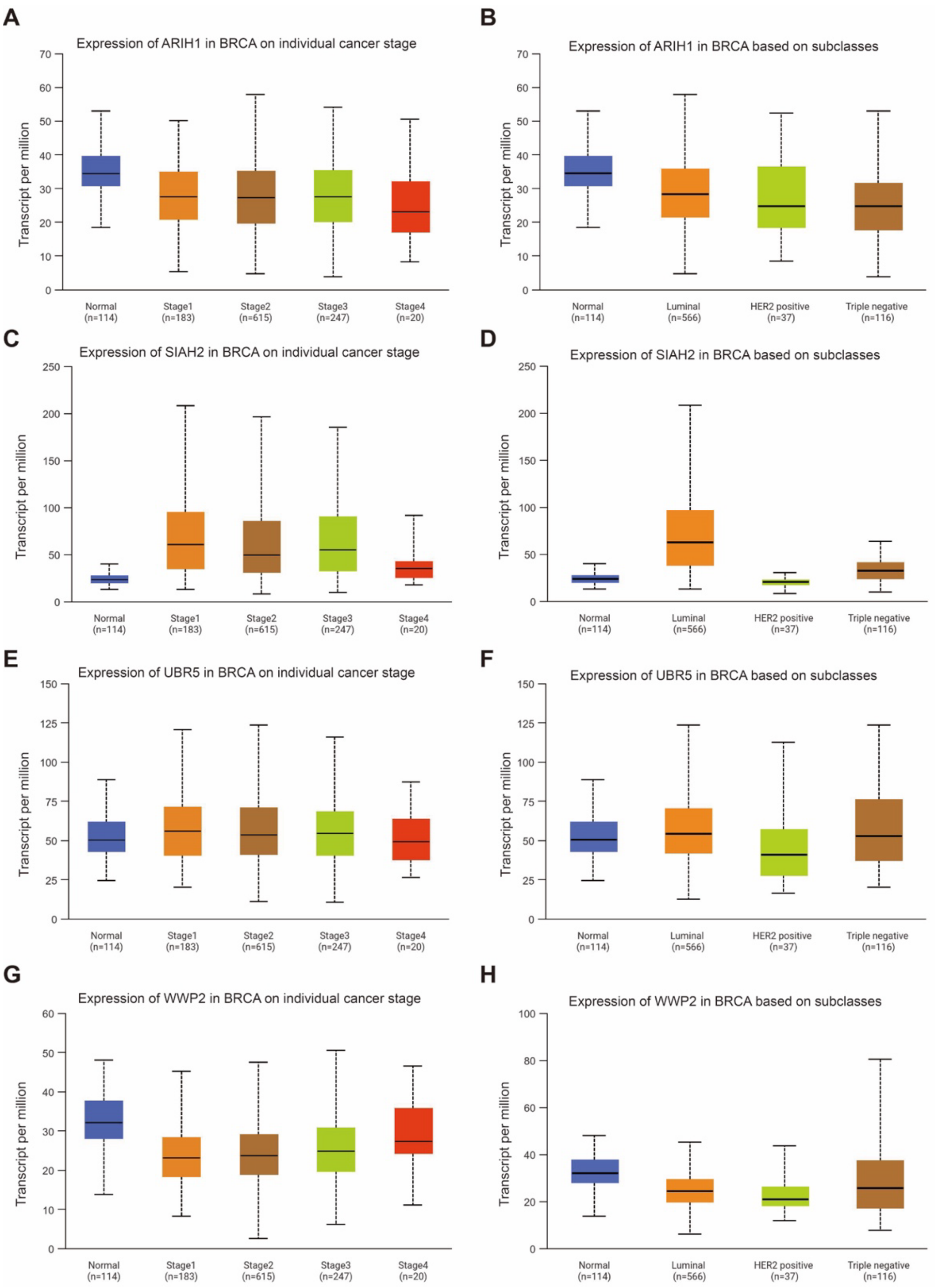


**Figure S3.** Expression levels of ARIH1, SIAH2, UBR5, and WWP2 across breast cancer stages and molecular subtypes. (A, C, E, G) Significant differences were observed for ARIH1 (Normal-vs-Stages 1, 2, 3, 4; $p < 0.05$), SIAH2 (Normal-vs-Stages 1, 2, 3, 4; $p < 0.05$), UBR5 (Normal-vs-Stages 1, 2, 3; $p < 0.05$), and WWP2 (Normal-vs-Stage 2, 3; $p < 0.05$). (B, D, F, H) Significant differences were found for ARIH1 (Normal-vs-Luminal, Normal-vs-TNBC; $p < 0.05$), SIAH2 (Normal-vs-Luminal, Normal-vs-TNBC, Luminal-vs-HER2 Positive, Luminal-vs-TNBC, HER2 Positive-vs-TNBC; $p < 0.05$), UBR5 (Normal-vs-Luminal, Normal-vs-TNBC, Luminal-vs-HER2 Positive; $p < 0.05$), and WWP2 (Normal-vs-Luminal, Luminal-vs-TNBC, HER2 Positive-vs-TNBC; $p < 0.05$). Statistical significance was determined by $p < 0.05$.

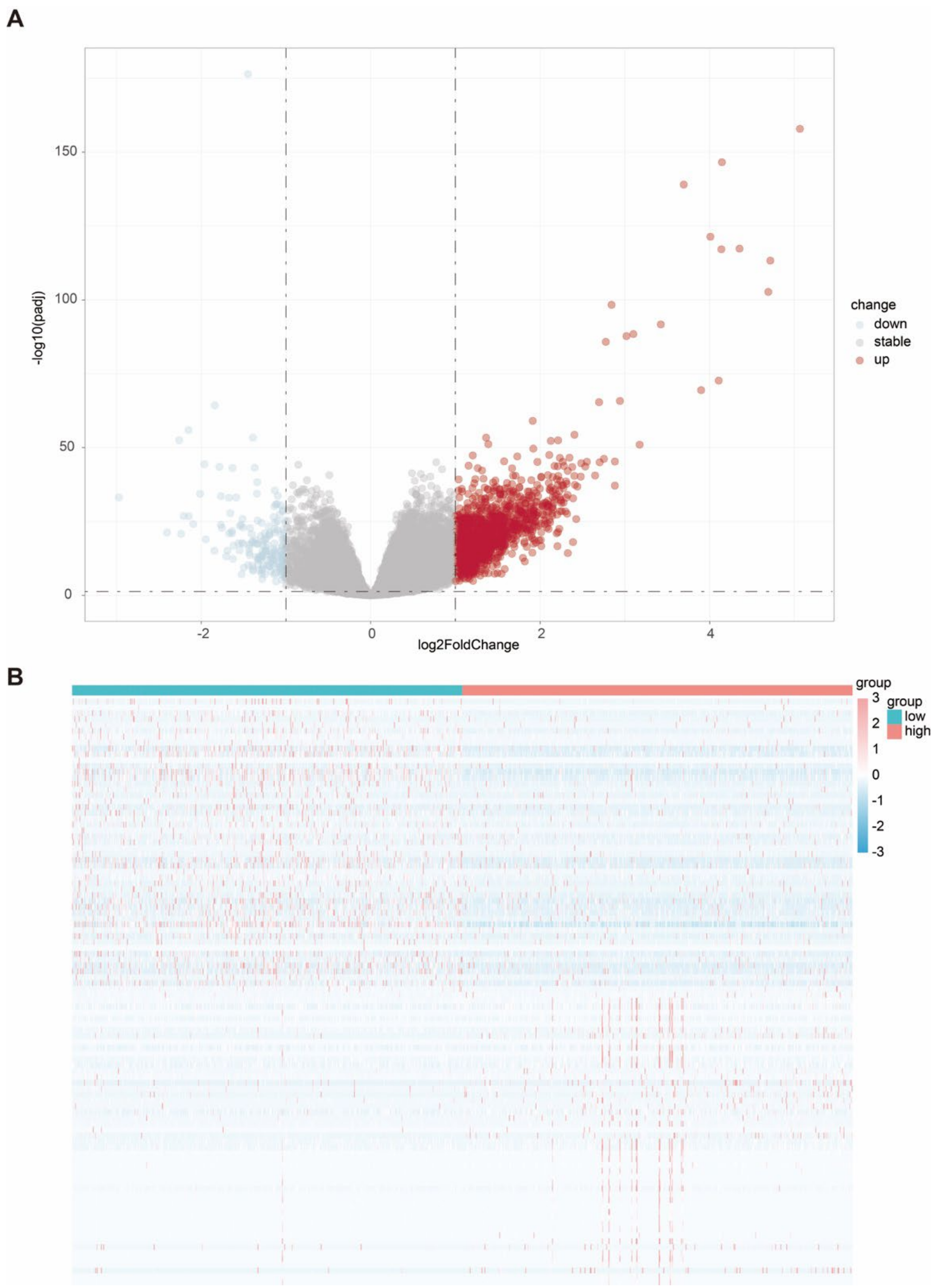


**Fig S4.** Differentially gene expression analysis between two groups. (A) Volcano plot depicting the DEGs between high- and low-risk groups according to the ASUW model. Genes were filtered using criteria of |log2FoldChange| > 1 and p-value < 0.05. Upregulated genes in the high-risk group are shown in red, downregulated genes are shown in blue, and non-significant genes are represented in gray. (B) Heatmap illustrating the expression profiles of the top 50 upregulated and 50 downregulated DEGs in the high- and low-risk groups. Rows represent individual genes, and columns represent patient samples. The heatmap colors indicate gene expression levels (red for upregulation and blue for downregulation). High-risk samples are marked in red, and low-risk samples are marked in blue.

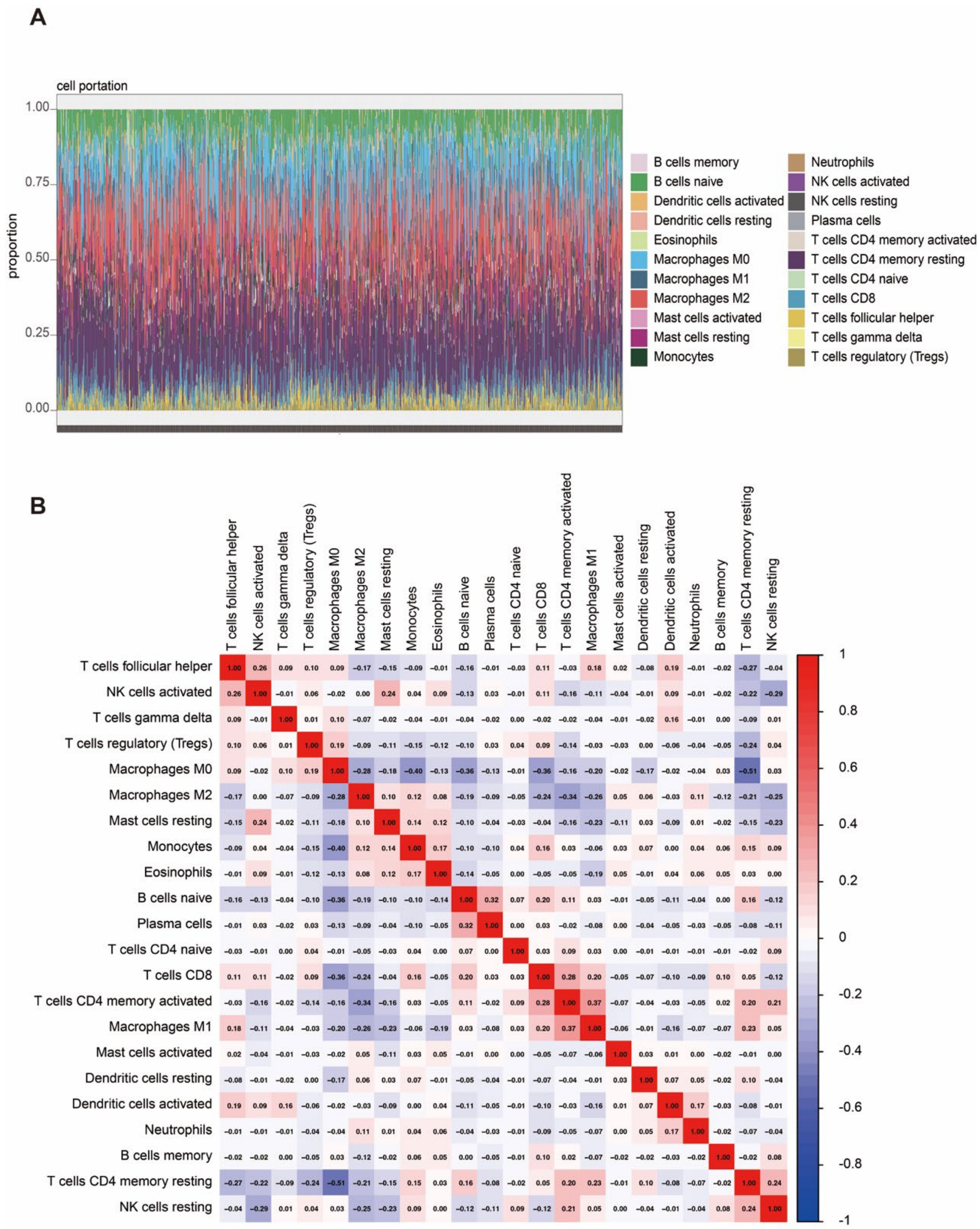


**Figure S5.** Proportion and correlation analyses of immune cells in BRCA patients (A) The composition of immune cells in breast cancer patients was analyzed using CIBERSORT, showing the proportional distribution of 22 types of immune cells across all samples. Each bar represents an individual patient, with different colors corresponding to distinct immune cell types. (B) Heatmap illustrating the correlation coefficients between different immune cell types in breast cancer patients. Positive correlations are indicated by red, while negative correlations are indicated by blue. The strength of the correlation is represented by the intensity of the color.
